# The Impact of Sales Volume and Limited Quantity on Intertemporal Choice in an Online Consumption Context

**DOI:** 10.3390/bs13070573

**Published:** 2023-07-11

**Authors:** Dawei Wang, Jiahui Li, Qiong Wu, Huiyan Li, Yixin Hu

**Affiliations:** School of Psychology, Shandong Normal University, Jinan 250061, China; wangdw@sdnu.edu.cn (D.W.); 2021020249@stu.sdnu.edu.cn (J.L.); 2021020240@stu.sdnu.edu.cn (Q.W.)

**Keywords:** online consumption, intertemporal choice, sales volume, limited quantity

## Abstract

In the context of online consumption, consumers are often faced with a decision between buying now or later. This study examines intertemporal choice by conducting two mixed experimental designs on a total of 206 college students to investigate the impact of combining sales volume and limited quantity on online consumption decisions. Experiment 1 revealed that under unlimited conditions, the same option with high sales volume was relatively more attractive and participants were more inclined to choose it, leading to a herding effect. However, under limited conditions, there was no scarcity effect. Experiment 2 built upon the findings of Experiment 1 and added dynamic change in sales volume. It was found that even with dynamic changes in sales volume, high sales volumes could still lead to a herding effect. In the case of unlimited conditions, dynamic changes in sales offset the effect of sales volume on intertemporal choice. Sales volume and limited quantity are important factors that influence consumers’ purchasing decisions. Therefore, this study combines sales volume and limited quantity and adds dynamic changes in sales to explore individuals’ intertemporal choices in online consumption situations. The findings of the study have significant implications for both merchants and consumers.

## 1. Introduction

Intertemporal choice refers to the process by which individuals evaluate the gains or losses at different points in time and make decisions [1,2,3,4]. Decision makers are faced with the dilemma of choosing between an option that offers immediate but smaller rewards (Small-Sooner, SS) and another option that provides larger rewards but requires waiting for a certain period (Larger-Later, LL). In recent years, with the rapid growth of the Internet economy, such intertemporal choice has become increasingly prevalent in online situations. Intertemporal choice in online consumption contexts refers to consumers’ decisions between immediate and delayed promotions when making purchases on the internet [5]. To illustrate, during the most prevalent “online promotion festival”, merchants issue coupons larger than usual in advance for use on the day of the promotion festival. Therefore, in order to attract a large number of potential consumers to the sale, creating a “delayed promotion”. Before the promotion festival, the merchants will inform the activity content in advance of “warm-up”, so that consumers can fully understand the products during this period of “delayed promotion” preferential activities. This allows for advanced preparation before the actual promotion festival [6]. At this point, consumers’ purchase decisions lead to differences in the timing of their access to products, inadvertently creating an intertemporal choice—“buy early and enjoy” or “wait for a lower price”?

As online consumption continues to grow, intertemporal choice in online consumption situations is becoming more and more prevalent. There are many antecedents that influence online consumption decisions, such as product reviews and promotions [7]. And intertemporal choice in online consumption situations usually occurs in the specific context of promotions. Promotions are utilized by merchants to incentivize consumers to purchase and benefit financially from them, which are conducted in a variety of ways to attract consumers to purchase [8,9]. Merchant promotions can be categorized into two main forms: general promotions and restricted promotions.

General promotions are those without quantity or time limitations. In this case, consumers may lack sufficient information to make informed choices, leading to a herding effect. Researchers initially discovered this herding effect in stock investment decisions, whereby investors imitate other investors in the trading process and buy the same stocks [10]. Chen also revealed the presence of herding effect in online consumer contexts [11]. Overall, 80% of online product sales were influenced by the displayed information about the products. For example, the historical sales information of the store has a significant positive effect on consumers’ selection of the store [12,13]. Consumers can infer product quality by observing the decisions made by previous purchasers and are influenced by others’ decisions when making their own purchase choices. Therefore, sales volume serves as a cue that triggers herd behavior and can significantly impact consumers’ consumption decisions [14,15].

Restrictive promotions refer to the restriction of purchase conditions on consumers during promotions, which are mainly divided into quantity restrictions and time restrictions [16]. Quantitative restrictions limit the number of promotional products that consumers can purchase (either in total or per transaction). Time constraints limit the period during which consumers can purchase promotional products. Time pressure significantly influences purchase decisions, and the strength of the time restriction can exert varying degrees of pressure on consumers, resulting in different effects on their purchase decisions [17,18]. Such quantitative and time constraints contribute to a sense of scarcity. According to Brock’s theory of commodities, scarcity increases the value of anything that can be owned [19]. The rarer a good is, the more valuable it becomes, and limited promotions enhance perceived scarcity [20]. Scarcity creates a “scarcity mindset” that increases attention to the scarce resource itself, causing individuals to constantly weigh different needs, resulting in varied decisions and behaviors [21,22,23]. Therefore, limited quantity could also influence consumers’ consumption decisions.

There are some innovative aspects of our study. Firstly, the combination of online consumption and intertemporal choice is an emerging field; there have been relatively few studies conducted in this area. This study aims to investigate intertemporal choice in the context of online consumption, with a focus on its underlying mechanisms and implications. Secondly, this study is presented in the context of a large online promotional shopping festival, where it is particularly important for consumers to make rational decisions in this frenzied shopping atmosphere. Finally, previous studies have underestimated and overlooked the role of dynamic changes in sales volume, which can reflect a dynamic growth trend in the real context of online shopping. Therefore, this study incorporates dynamic changes in sales to investigate the impact of combining sales volume and limited quantity on individual intertemporal choice in online consumption.

## 2. Literature Review and Hypotheses

Online consumption is full of uncertainty. Before purchasing products, consumers tend to assess the various risks associated with the transaction. The uncertainty and potential negative consequences perceived by consumers when purchasing a product could be transformed into their perception of risk. The virtual nature of the Internet, concerns over credit card information security, product quality, whether it is convenient to unsubscribe and other factors will deepen the risk perception of consumers in the process of online shopping. In the online shopping environment, consumers perceived a higher level of risk compared to traditional shopping [24]. Therefore, in the online environment, consumers actively seek product-related information to reduce shopping uncertainty, and product sales volume provided a reference value for consumer decision-making. In the context of online consumption, consumers with a higher level of risk perception tended to rely more on herd cues when predicting product quality [25]. Consequently, we hypothesized that the online consumption environment might increase the uncertainty of products when faced with the intertemporal choice situation, so that consumers were likely to rely more on sales information. When delayed promotions resulted in higher sales, consumers would show a herding effect and choose delayed promotions (LL) more often. Consumers would extract cues about the popularity of a product from the information displayed, of which the number of coupons received could be considered one such information cue. This study speculated on the effect of sales volume on intertemporal choice, which could be approached from the perspective of the number of coupons received. The greater the number of coupons claimed by consumers, the higher their perceived sales volume for that particular product. Therefore, we proposed that:

**Hypothesis** **1.** *The main effect of sales volume is significant. The higher the sales volume of the LL option, the more frequently participants choose the LL option, and herding effect appears*.

A scarcity mindset could result in a decline of individual cognitive ability, weakening the capacity for analysis, judgment and logical reasoning. At the same time, it might lead to reduced executive control and impaired ability to inhibit behavior and control impulses [26]. Research in decision neuroscience further confirms the existence of a core network for subjective evaluation of rewards from different domains, including areas of the ventral striatum (VS) and orbitofrontal cortex (OFC). The increase of OFC activation predicts increased subjective valence [27]. Huijsmans et al. found that the scarcity mindset affects neural mechanisms associated with consumer choice, and participants under scarcity mindset have increased activation in the orbitofrontal cortex [28]. These findings provided support for the notion that scarcity could directly influence decision-making processes based on goal-directed values. Therefore, we hypothesized that scarcity mindsets triggered by limited conditions (quantity) could influence intertemporal choice under online consumption, resulting in reduced cognitive abilities and decreased selection of delayed promotions (LL) under limited conditions. We hypothesized that:

**Hypothesis** **2.** *The main effect of limited quantity is significant. Compared with the not limited quantity group, the limited quantity group will reduce participants’ choice of delayed option (LL)*.

One study found that a limited quantity does not always lead to higher purchase intentions, and its effectiveness depends on other factors [29]. He et al. found that information on sales volume levels and limited quantity levels could be used as external cues when consumers made choices about online products [30]. In real life situations, relative scarcity due to insufficient stock (i.e., limited quantity) and product popularity (i.e., high sales volume) is very common [31]. A limited number of products (limited quantity) increases the perception of a high demand product (high sales volume), increasing consumers’ purchase intention [32]. It refers to the consumer’s willingness to pay for a product in the future and serves as an indicator of their attitude towards the product [33]. In a consumption context, consumers’ purchase intention is a prerequisite for making consumption decisions. Moreover, it has been shown that consumers infer the quality of a product based on the scarcity associated with high sales volume and thus decide to purchase the scarce product, from which could create the herding effect [34]. The scarcity effect is greater when more consumers have already purchased the scarce product. There is a study that found that the informational tendency of sales volume levels dominates when both sales volume levels and limited quantity levels are present [31]. Therefore, we hypothesized that the combination of sales volume and limited quantity would have an impact on intertemporal choice in online consumption contexts. We hypothesized that:

**Hypothesis** **3.** *The interaction between sales volume and limited quantity is significant. In the case of high sales volume, compared with the not limited quantity group, participants in the limited quantity group are more likely to choose the delayed option (LL)*.

In the context of online consumption, sales volume is dynamic rather than static. People prefer outcomes with an increasing trend over stable or decreasing trends. Dynamic increases in sales volume significantly affect the likelihood of purchase and even reverses consumer preferences for products with higher sales rankings [12]. Furthermore, studies on intertemporal choice have shown that people favor a range of improved outcomes [35]. Therefore, we hypothesized that participants might prefer this option when making intertemporal choices in the context of online consumption. Studies have shown that external cues such as sales volume and limited information significantly influence consumers’ online product choices, with sales volume playing a more dominant role between the two factors [31]. Based on the above arguments, for one thing, we contend that individuals tend to rely on sales volume information and opt for high sales options when presented with both sales volume and limited quantity data simultaneously. For another, when sales volume increases dynamically, individuals might ignore whether the products are limited or not and prefer options with dynamic changes. Therefore, we presented the following hypotheses:

**Hypothesis** **4.** *The main effects of sales volume and limited quantity and the interaction effect between the two are still significant after the addition of dynamic change of sales*.

**Hypothesis** **5.** *The main effect of dynamic change of sales is significant. Compared with static sales, participants prefer the option of a dynamic increase in sales volume*.

**Hypothesis** **6.** *The interaction between sales volume, limited quantity and dynamic change is significant*.

In summary, this study proposed two experiments to explore the effect of sales volume and limited quantity on intertemporal choice in an online consumption context. Experiment 1 explored the effect of sales volume (high, medium and low) and limited quantity (limit vs. no limit) on consumers’ intertemporal choice when sales volume was static in an online consumption context. To better represent this effect, Experiment 1 used option attractiveness scores and the frequency of LL option as dependent variables. Attractiveness was measured using a nine-point Likert scale. In order to fit the dynamic change process of sales volume in a real online consumption context, Experiment 2 further compared the intertemporal choices when sales volume was stationary and when it increased dynamically. The aim was to investigate whether the dynamic change in sales volume had any effect on consumers’ intertemporal choices in an online consumption context, and whether this effect differed from that found in Experiment 1.

## 3. Experiment 1: The Effect of Sales Volume and Limited Quantity on Intertemporal Choice in an Online Consumption Context

Experiment 1 aims to explore the impact of different sales volume and limited quantity on individuals’ option attractiveness scores and intertemporal choice in online consumption contexts.

### 3.1. Method

#### 3.1.1. Participants

The G*Power software (version 3.1) was used, and the appropriate sample size to detect a moderately-sized effect (effect size *f* = 0.25, α = 0.05, 1 − β = 0.8) was at least 86 participants. In Experiment 1, a total of 124 participants (*M*_age_ = 19.78 years, *SD* = 1.68; 28 males and 96 females) were selected from enrolled college students using a random sampling method. There were no invalid participants. The study was approved by the Academic Committee of Shandong Normal University and followed the Declaration of Helsinki. Participants were voluntary and anonymous, signed a written informed consent form, and had the right to withdraw from the experiment at any time. All participants were in good health and were given a gift as a reward for completing the experiment.

#### 3.1.2. Experimental Design

A two-factor mixed experimental design of 3 (sales volume: LL high, LL medium, LL low) × 2 (limited quantity: limit, no limit) was used in this study, with sales volume as a within-subject variable and limited quantity as a between-subject variable. In the intertemporal choice task, the dependent variable was the frequency (f) of choosing the LL option at different sales volume levels. To further test participants’ choices, attractiveness ratings were used as the dependent variable to measure the difference in attractiveness of the different options to participants. In the attractiveness task, the dependent variable was the relative attractiveness of the LL option, i.e., the difference between the attractiveness score of the LL option and the SS option (D), which is calculated as D = Score(LL) − Score(SS).

#### 3.1.3. Experimental Materials

**Figure A1:**
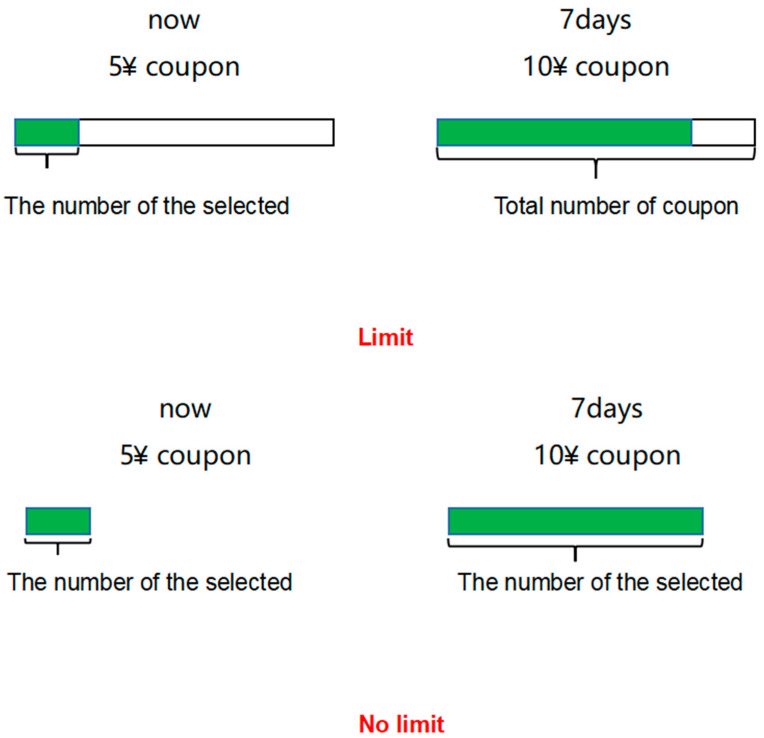
Two examples of the intertemporal choice task in online consumption.

Pyone used “instant rebate” and “delayed rebate” as two options for intertemporal choice in his research [5]. This experiment referred to the study and changed the form of rebates to coupons, with the addition of a shorter delay period. The coupons can be used now or delayed. The former had a small and fixed amount, while the latter had a large amount with varying delay time and amount (hereinafter referred to as SS option and LL option). In order to facilitate the understanding of the participants, “selected number” represented the sales volume, “total amount” represented the upper limit of the quantity of the coupon. The length of the quantity bar (in green) below corresponded to the sales volume, and the black border of the quantity bar in the limited quantity group meant that the total number of coupons was limited (see Figure A1). It had been suggested that time and value were two important variables to consider when predicting consumer choice of coupons and this adaptation could model intertemporal choice in the context of online consumer promotions [36]. The experiment utilized those materials and required participants to evaluate and choose between a fixed SS option and a changing LL option for each choice.

This study was programmed on E-prime 2.0. The SS option was a $5 coupon available now, while the LL option was set in increments of 50%, 100%, 150% and 200% for four amounts ($7.50, $10, $12.50, $15) and three delays (7 days, 14 days, 28 days) to approximate the actual online shopping situation, e.g., “$10 coupon available after 7 days”. They could be combined to produce 12 such choice pairs, which mixed with the three sales volume levels produced a total of 36 trials. Thus, participants needed to perform 72 ratings in the attractiveness task and complete 36 trials in the intertemporal choice task. In the attractiveness task, coupons were rated on a scale of 1 to 9, with 1 representing “low attractiveness” and 9 representing “high attractiveness” [37].

During the study, both experimental tasks had no time limit. After each trial, “+” would appear in the center of the screen, which would continue for 500 ms and automatically enter the next trial.

#### 3.1.4. Procedure

The experiment was completed in a quiet laboratory. Participants were randomly assigned to either a limit or no limit group. Each participant completed the option attractiveness scoring task and the intertemporal choice task in all three sales conditions, and the order of the two tasks was balanced in the experiment. All trials were presented randomly.

Participants were asked to fill in demographic variables before the experiment began. The experimenter then explained the context of the experiment and explained the attractiveness scoring task (or intertemporal choice task) to participants in different groups (limit and no limit groups). At the end of the practice phase, the participants were prompted with the text “Practice is over, press E to start the formal experiment”. There was a 2-min break between the two experimental tasks. Finally, we thanked the participants and gave them small gifts as a reward for their participation.

### 3.2. Results

The main effects of demographic variables, such as gender and age, were not found to be significant in the study. Therefore, they were not detailed in the data analysis.

**Figure 1:**
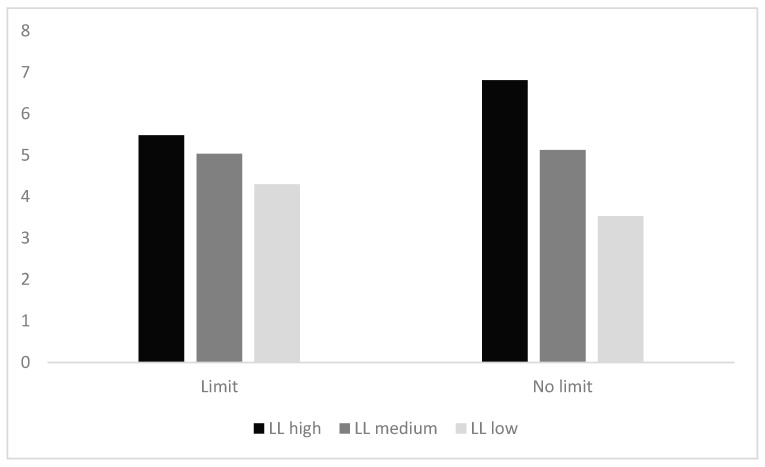
Frequency Diagram. Notes: The two groups of participants selected the LL option frequency chart at different sales volume levels.

First, the participants pressed the “J” button at each sales volume level in both limit and no limit groups. The frequency (f) of choosing the LL option (as shown in Table 1) was recorded, and a line graph of its frequency change was drawn (as shown in Figure 1). Each sales level contained 12 trials, so f ranged from 0 to 12, with f > 6 indicating a relatively greater preference for the LL option and f < 6 indicating a relatively greater preference for the SS option.

A repeated-measures ANOVAs on the two groups of participants showed a significant main effect of sales volume. There was a significant difference in participants’ choices at different levels of sales volume, *F* (2, 244) = 39.359, *p* < 0.001, η_p_^2^ = 0.244, *M*_LL high_ = 6.17, *M*_LL medium_ = 5.08, *M*_LL low_ = 3.90. Post hoc multiple comparisons showed that participants in the LL high group (*M* = 6.15, *SD* = 0.28) chose the LL option significantly more often than the LL medium group (*M* = 5.08, *SD* = 0.24) and the LL low group (*M* = 3.92, *SD* = 0.24), *p*s < 0.001. The main effect of limited quantity was not significant, and there was no significant difference between participants’ choices in the limit and no limit groups (*p* > 0.05). The interaction effect between limited quantity and sales volume was significant, *F* (2, 244) = 8.779, *p <* 0.001, η_p_^2^ = 0.067.

Due to the significant interaction effect of sales volume and limited quantity, a further simple effects analysis of this interaction was conducted. The results found that for the limit group, there was no significant difference (*p* > 0.05) in participants’ choices at different sales volume levels (*M*_LL high_ = 5.48, *M*_LL medium_ = 5.03, *M*_LL low_ = 4.30). For the no limit group, there were significant differences in participants’ choices at different sales volume levels. Higher sales volumes of the LL option were associated with a lower propensity to choose the SS option and an increase in the frequency (f) of choosing the LL option (*M*_LL high_ = 6.81, *M*_LL medium_ = 5.13, *M*_LL low_ = 3.53).

**Figure 2:**
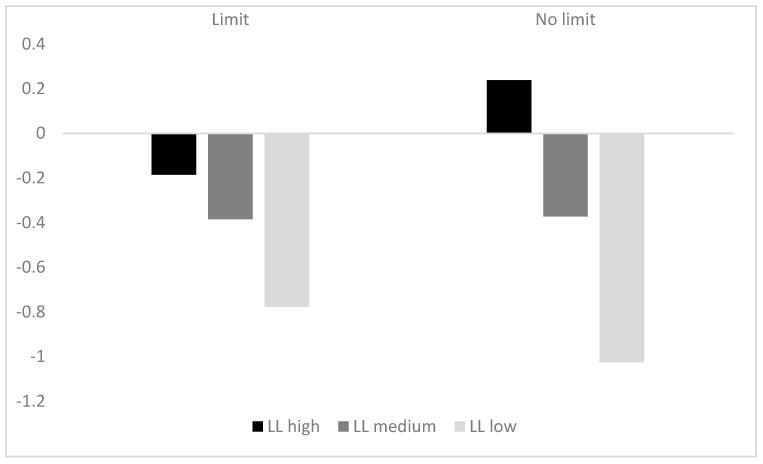
Attractiveness Rating Difference.

The difference (D) between the participants’ ratings of the attractiveness of the LL option and the SS option at different sales volume levels were counted (as shown in Table 2), and a line graph of the change in difference (D) was made (as shown in Figure 2).

A repeated-measures ANOVAs revealed a significant main effect of sales volume. A significant difference in the relative attractiveness of the two options to participants at different levels of sales volume, *F* (2, 244) = 21.272, *p* < 0.001, η_p_^2^ = 0.148, *M*_LL high_ = 0.03, *M*_LL medium_ = −0.38, *M*_LL low_ = −0.90. Post hoc multiple comparisons revealed that the LL high group (*M* = 0.03, *SD* = 0.21) was significantly more attractive to participants than the LL medium group (*M* = −0.38, *SD* = 0.18) and the LL low group (*M* = −0.90, *SD* = 0.21), *p*s < 0.001. The main effect of limited quantity was not significant (*p* > 0.05). At the same time, the interaction effect between limited quantity and sales volume was marginally significant, *F*(2, 244) = 2.933, *p* = 0.055, η_p_^2^ = 0.023.

### 3.3. Discussion

The results of Experiment 1 revealed a significant main effect of sales volume. Participants in both the limit and no limit groups were more inclined to choose the delayed option when LL sales volume was high, and to choose the immediate option when LL sales volume was low, compared to the medium LL sales volume. When LL sales volume was high, participants had an increased tendency to choose the LL option, which made more choices that were consistent with the majority of the previous consumers. As a result, it could be assumed that a herding effect occurred, which further corroborated Hypothesis 1. Specifically, at medium LL sales volume, the SS option was more attractive to participants and the LL relative attractiveness was negative. However, when LL sales volume was high, participants’ attractiveness rating difference (D) increased at different sales volume levels, i.e., LL’s relative attractiveness increased, and participants were more willing to wait for larger coupons. When LL sales volume was low, participants’ attractiveness rating difference (D) decreased at different sales volume levels, i.e., LL’s relative attractiveness decreased, and participants were more willing to choose smaller coupons for immediate use.

The main effect of limited quantity was not significant. Specifically, there was no difference in selection between the limited and unlimited groups, which was contrary to Hypothesis 2. The possible reasons and explanations are as follows. First, the experiment was conducted in a virtual online shopping environment, which did not consider the participants’ actual purchase demand for hypothetical products. Therefore, consumers were not as sensitive to scarcity information when browsing online as they are when actually shopping, which might affect the validity of scarcity information [20]. Second, the participants selected for the experiment were college students who were more experienced with online shopping, and they may be less affected by the scarcity of products. Third, both the SS and the LL options were limited, which indicated that scarcity existed for both options, and participants may accordingly not have perceived the difference in option scarcity. Thus, resulting in no significant difference in participants’ choices when limited versus unlimited conditions.

The interaction between sales volume and limited quantity was marginally significant. In the not limit group, participants’ choices at different sales volume levels remained significantly distinct. Compared to medium sales volume, participants chose the option more frequently at high LL sales volume and less frequently at low LL sales volume. However, in the limited group, participants’ choices did not significantly differ across sales volumes. The relatively greater scarcity of coupons with high sales volume compared to low sales volume did not increase participants’ propensity to choose the high-sales volume option, which was inconsistent with Hypothesis 3. It is speculated that the reason for this might be related to psychological resistance [38]. The limit caused resistance among participants in making a choice, leading to an increased willingness to choose the high-sales volume option to neutralize. As a result, even though the scarcity was higher, the high-sales volume option did not receive a stronger preference.

## 4. Experiment 2: The Impact of Sales Dynamics on Intertemporal Choice in an Online Consumption Context

The objective of Experiment 2 is to examine the impact of dynamic sales volume on intertemporal choice in online consumption situations, as well as to investigate potential herding and scarcity effects.

### 4.1. Method

#### 4.1.1. Participants

The G*Power software (version 3.1) was used, and the appropriate sample size to detect a moderately-sized effect (effect size *f* = 0.25, α = 0.05, 1 − β = 0.8) was at least 72 participants. In Experiment 2, a total of 82 participants (*M*_age_ = 19.48 years, *SD* = 0.69; 20 males and 62 females) were selected from enrolled college students using a random sampling method. There were no invalid participants. The study was approved by the Academic Committee of Shandong Normal University and followed the Declaration of Helsinki. Participants were voluntary and anonymous, signed a written informed consent form and had the right to withdraw from the experiment at any time. All participants were in good health and were given a gift as a reward for completing the experiment.

#### 4.1.2. Experimental Design

A mixed experimental design of 3 (sales volume: LL high, LL medium, LL low) × 3 (sales dynamic changes: stationary, SS sales volume increase, LL sales volume increase) × 2 (limited quantity: limit, no limit) was used in this study, with sales volume and sales dynamic changes as within-subject variables and limited quantity as between-subject variable. This experiment only had an intertemporal choice task and the dependent variable was the frequency with which participants chose the LL option under each experimental treatment (f).

#### 4.1.3. Experimental Materials

Experiment 2 still used the intertemporal choice task and added two types of sales volumes that change dynamically (SS sales volume increase, LL sales volume increase) to the stationary sales volume of Experiment 1. Participants in the limit and no limit groups completed intertemporal choice tasks of nine different treatments, respectively. Among these treatments, six included dynamic changes while three were static. Each experimental treatment still consisted of 12 trials (the same as Experiment 1), and each participant needed to complete 108 trials in total.

#### 4.1.4. Procedure

This study was essentially the same as Experiment 1 and required several participants to complete it simultaneously in the same quiet laboratory. Participants were randomly assigned to either the limit or no limit group and each participant was asked to complete a series of intertemporal choice tasks under a total of 3 (sales volume: LL high, LL medium, LL low) × 3 (sales dynamic changes: stationary, SS sales increase, LL sales increase) conditions. Several participants balanced the order of the two experimental tasks, stationary (36 trials) and dynamic change (72 trials). During the experiment, with a 2-min break between the two tasks and a random presentation of the trials in each task. The experimental procedure was the same as in Experiment 1.

### 4.2. Results

The main effect of demographic variables, such as gender and age, were not found to be significant. Therefore, they were not detailed in the data analysis.

Firstly, the frequency of selecting the LL option (f) was separately counted for each treatment in both limit and no limit groups, based on the number of times the participant pressed the “J” key (as shown in Table 3).

A repeated-measures ANOVAs of 3 (sales volume: LL high, LL medium, LL low) × 3 (sales dynamic changes: stationary, SS sales increase, LL sales increase) × 2 (limited quantity: limit, no limit) was conducted and showed that there was a significant main effect of sales volume after adding the sales dynamic as a within-subject variable, *F* (2, 320) = 11.108, *p* < 0.001, η_p_^2^ = 0.122. The main effect of limited quantity and sales dynamic changes was not significant (*p*s > 0.05). The interaction effect of any two of limited quantity, sales volume and sales dynamic changes were not significant (*p*s > 0.05). However, the interaction of all three were significant, *F* (4, 320) = 2.726, *p* = 0.029 < 0.05, η_p_^2^ = 0.033.

Further simple effects tests on the three independent variables revealed significant differences in the frequency of choosing the LL option between the different sales volume levels of the limit group under sales dynamic change conditions. As evidenced by the fact that the LL medium group (*M* = 6.28, *SD* = 0.51) was significantly higher than the LL low group (*M =* 5.40, *SD* = 0.49) under stationary conditions; the LL high group (*M* = 6.33, *SD* = 0.49) was significantly higher than that of the LL low group (*M* = 5.15, *SD* = 0.45) under SS sales dynamic changes group; the LL high group (*M* = 6.60, *SD* = 0.52) was significantly higher than that of the LL low group (*M* = 5.20, *SD* = 0.46) under LL sales dynamic changes group, *p*s < 0.05. The no limit group differed significantly between different sales levels only in the stationary state. Concretely speaking, the LL high group (*M* = 6.64, *SD* = 0.50) was significantly higher than both the LL medium group (*M* = 6.05, *SD* = 0.50) and the LL low group (*M* = 4.86, *SD* = 0.48), *p*s < 0.01.

### 4.3. Discussion

The findings of Experiment 2 revealed that the main effect of sales volume remained significant, as individuals exhibited a herding effect when the sales volume of LL was high. Conversely, the main effect of limited quantity was not significant, which contradicted Hypothesis 4 but aligned with Experiment 1’s results. The main effect of sales dynamic changes was not significant, indicating that such changes could not change participants’ choice preferences or enhance their propensity to choose a particular option. This finding was inconsistent with Hypothesis 5 and could be explained by the following reasons. Firstly, it might be that the magnitude of the sales dynamic changes was too low and the sales volume remained at a low level before the change, which was insufficient to prompt participants to alter their preference choices in the stationary state. Furthermore, the change might be too small to attract participants to cause a difference in their choice.

The interaction between limited quantity and sales volume was not significant, indicating that the scarcity due to high sales volume and limit did not result in a significant scarcity effect. However, the interaction between sales volume, limited quantity and the sales dynamic changes were significant, which was consistent with Hypothesis 6. Further simple effects analysis revealed a significant difference (*p* < 0.05) between the no limit group and different sales volume levels at static, consistent with the findings of Experiment 1. However, with the addition of sales dynamic changes, there was no significant difference in participants’ choices at different levels of sales volume. When the sales volume changed dynamically, two scenarios emerged: SS sales increase and LL sales increase. We took the dynamic increase in SS option sales volume as an example to analyze the reasons for this phenomenon. If sales volume of both LL and SS options were low, an increase in SS sales would widen the gap between the two options. This could lead participants to doubt the authenticity of this increase and speculate that the merchant deliberately created extreme differences to push consumers towards choosing the SS option, thus creating resistance. However, high sales volume would still increase participants’ willingness to choose the SS option. This conflict might moderate the conflicting desires of the participants and lead them to make the same choice as in the medium sales volume. If the LL option had high sales volume, the dynamic growth of SS option would narrow the difference between the two options, increasing willingness to choose SS, while also increasing willingness to choose LL. There was no significant difference between the two choices and those with medium sales volume. The dynamic growth of LL option sales volume could be similarly deduced. At this point, the effect of sales volume was offset by sales dynamic changes, resulting in no significant difference in participants’ choices at different sales volume levels.

## 5. General Discussion

This study examined the effect of sales volume and limited quantity on intertemporal choice in the context of online consumption, and further analyzed how this impact changes with dynamic changes in sales. Previous studies traditionally considered sales volume as a dependent variable that required prediction by other factors [39,40,41], with few examining it as an independent factor influencing consumers’ purchase intentions. In contrast to previous studies, this study used sales volume as a predictor variable and created an intertemporal choice situation of “buy early and enjoy” or “wait for lower prices” by using coupons from merchants’ promotions.

In Experiment 1, high sales volume resulted in a herding effect, and in Experiment 2, this effect persisted when sales dynamic changes were added. The herding effect of high sales volume could be attributed to both informational and normative influences [42]. Informational influence refers to the phenomenon where individuals seek guidance from others in uncertain situations and make their own decisions with reference to the behavior of others. In the present study, this was exemplified by participants perceiving sales volume as a reflection of others’ choices. Therefore, their own decisions could be influenced by others to conform with them, resulting in the phenomenon of herding. Normative influence refers to the social pressure that individuals experience in order to avoid rejection or gain acceptance from others, and overt purchasing behavior is particularly susceptible to this type of influence. Participants in this study assumed that their choices would be reflected in sales volume and might have developed evaluative scruples [43]. In summary, the participants’ preferences and evaluations may have been influenced by informativeness and normativity, leading to changes in decisions and a herding effect. The herding effect from high sales volume remained when sales dynamic changed, yet the differences observed in participants’ choices between the stationary state and the two sales dynamic changes were weak and not significant. The reason for this was that the experiment was set up in a virtual consumer choice scenario, where participants were informed that they were not alone in making their choices about coupons. This created a sense of social presence, which refers to the feeling of warmth and social interaction with others who are also shopping online at the same time as the consumer [44]. According to the theory of social facilitation, the presence of others had a social arousal effect and influenced individuals’ attitudes and behaviors [45]. Given that, if the online environment provided consumers with a heightened sense of presence, they were more likely to engage with it. The information (sales dynamic changes in the number of coupons selected) was amplified in their psychological perceptions [46]. However, sales dynamic changes set in this study were too low to foster a sense of interpersonal interaction among participants and thus did not trigger a sense of social presence.

A new finding was that the main effect of the limited quantity was not significant, regardless of whether sales dynamic changes were included or not. This could be explained in several ways. First, in online consumption, consumers exhibited less sensitivity to scarcity information compared to offline shopping, which might undermine the validity of scarcity information [20]. In addition, the participants were university students, who were generally more experienced with online consumption and might be less influenced by scarcity. Finally, the SS and LL options were both limited and both had scarcity, and participants did not perceive a difference in scarcity between the two options. There was no significant difference in participants’ choice between the limit and no limit conditions. In addition, the theory of psychological resistance could explain the fact that scarcity did not influence participants’ relative attractiveness and their choices. Psychological reactance refers to the aversive motivational states that individuals experience when their freedom is deprived or threatened [47], including both “trait resistance” and “state resistance”. The former refers to a stable personality trait, while the latter is a manifestation of intrinsic motivation elicited in a particular situation. There are three categories of reasons that account for consumers’ psychological resistance-perceived restrictiveness, perceived intrusiveness, and perceived operational intent [48]. The limited quantity scenario in this study resulted in supply scarcity, but consumers were suspicious of the merchant’s operational intentions in this scenario. Therefore, consumers perceived deceptiveness and evoked “state resistance”, which in turn led to negative evaluations of high-scarcity options (high sales volume, limit) and reduced their willingness to choose [49]. However, high sales volume increases participants’ willingness to choose options compared to low sales volume options. The neutralization of the two contradictory intentions resulted in no significant difference between participants’ choices under limited and not limited conditions. The main effect of limited quantity remained insignificant after the inclusion of change in sales dynamics. This could be explained by the following reasons. Firstly, the magnitude of the sales dynamic changes was too low to induce a change in participants’ perception of the scarcity of the changed option when the difference in sales volume between the two options was drawn closer. As a result, the perceived restriction did not change prominently and did not differ significantly from choices made under stationary conditions. Secondly, although participants perceived a change in scarcity, they were skeptical of the extreme differences that had been widened and believed it to be a “false scarcity” manipulated by the merchant. Subsequently, participants perceived increased intention to manipulate, a diminished level of trust and psychological resistance. Consequently, even if there had been a change in scarcity, it would not have caused a change in intertemporal choice [48].

Another finding was that the interaction between sales volume and limited quantity was marginally significant in Experiment 1, but not significant in Experiment 2 when sales dynamic changes were introduced. In addition, a triple interaction effect was observed among sales volume, limited quantity, and sales dynamic changes. In the limit group, there was no significant difference in the choice of each sales volume level across different stages of change. In the no limit group, there was a significant difference in selection at different sales volume levels in the stationary state. The above results are the same as Experiment 1, and Experiment 2 serves to complement its results. However, when sales volume dynamics changed, participants in the no limit group did not differ significantly in their choice of different sales volumes. This could be explained in real-life situations as follows: Firstly, high sales volume increased choice intention, but participants might have perceived a deliberate intention to create extreme disparities and increase the value of high-sales volume options in order to induce consumers to choose them when they dynamically increase. This could lead to a sense of psychological resistance due to the perceived deception. Secondly, the dynamic increase in the low sales volume option narrowed the gap between the two options and increased the willingness for it. The former canceled out the conflicting intentions of the high-sales volume option against each other, while the latter counteracted the increased choice intentions of both options. Thus, the coexistence of both dynamics neutralized the impact of sales volume, resulting in no significant differences in participants’ choices at each sales volume level.

## 6. Limitations and Future Directions

First, in terms of research methodology, the majority of studies suggested that scarcity increased consumers’ perceived value, positive attitude and evaluation towards a product, as well as their willingness to purchase—causing a scarcity effect [50]. One possible explanation for the failure of this study to demonstrate the scarcity effect was that the sales dynamics did not sufficiently alter participants’ perception of scarcity, resulting in no discernible differences in relative attractiveness or choice. In the future, it might be worthwhile to explore the impact of sales dynamic changes between different sales volume levels on participants’ evaluations and choices by increasing the magnitude of such changes, for instance from low to medium or high sales volumes.

Second, in terms of ecological validity, the scarcity manipulation did not trigger a scarcity effect in this study, which might not be consistent with the specifics of actual online consumption. Additionally, it should be noted that university students represented only a portion of the online consumer group and their intertemporal choice preferences might differ from other online consumers. College students generally have experience with online shopping, and conventional marketing tools cannot change their preferences choice. Moreover, the context of the intertemporal choice task might be too limited because participants might have different choices when faced with types of products other than clothing. In the future, we could consider variations in consumers’ intertemporal choices for diverse types of products (e.g., necessities versus luxury products, durable versus non-durable products, etc.).

Third, in terms of practical application, this study suggested that sales volume could cause a herding effect, which could yield positive outcomes for businesses and promote consumption. However, it remains challenging to discern the benefits and drawbacks for consumers. The future exploration of the psychological mechanisms underlying consumers’ herding effect in intertemporal choice within online consumption contexts was warranted. In addition, contextual influences alone might not fully account for the differences in intertemporal choice observed in online consumption contexts. Individual characteristics could also play a significant role in shaping consumer behavior.

## 7. Conclusions

This study examined the effects of combining sales volume and limited quantity on individual intertemporal choice in an online consumption context and explored the role of dynamic changes in sales volume. The following conclusions are drawn from this study: (1) Under the condition of no limit, when sales volume is high, the same option becomes relatively more attractive and participants are more inclined to choose it, resulting in a herding effect. (2) However, under the condition of limit, the scarcity caused by high sales volume does not significantly affect relative attractiveness and therefore does not produce a scarcity effect. (3) Even with high sales volume, changes in sales dynamics can still trigger a herding effect. (4) The interaction among sales volume, limited quantity, and sales dynamics changes is significant. Specifically, the sales dynamic changes in no limit condition counteract the effect of sales volume on participants’ intertemporal choices.

The findings of this study offer valuable insights for both consumers and merchants. For consumers, it is beneficial to remind them of the numerical pitfalls lurking behind merchants’ high sales figures. This will help reduce consumers’ blindness to follow the trend and encourage more rational online shopping behavior. By overcoming external factors that interfere with their decision-making process, consumers can make independent and informed choices. For merchants, they can enhance the appeal of their products by boosting sales volume, thereby stimulating consumers demand.

## Figures and Tables

**Table 1:** Experiment 1: Descriptive Statistics.

Group	LL High	LL Medium	LL Low
Limit *N* = 60	5.48 ± 3.25	5.03 ± 2.67	4.30 ± 2.82
No limit *N* = 64	6.81 ± 3.08	5.13 ± 2.59	3.53 ± 2.59

**Table 2:** Attractiveness Rating Difference.

Group	LL High	LL Medium	LL Low
Limit *N* = 60	−0.19 ± 2.14	−0.39 ± 1.93	−0.77 ± 2.23
No limit *N* = 64	0.24 ± 2.48	−0.37 ± 2.06	−1.03 ± 2.46

**Table 3:** Experiment 2: Descriptive Statistics.

Group		LL High	LL Medium	LL Low
Limit *N* = 40	Static	6.18 ± 3.40	6.28 ± 2.90	5.40 ± 3.15
	SS change	6.33 ± 3.10	5.65 ± 2.74	5.15 ± 2.83
	LL change	6.60 ± 3.47	6.00 ± 2.98	5.20 ± 2.97
No limit *N* = 42	Static	6.64 ± 3.09	6.05 ± 3.51	4.86 ± 3.06
	SS change	5.88 ± 3.04	5.38 ± 3.00	5.05 ± 2.80
	LL change	5.88 ± 3.09	5.90 ± 2.94	5.19 ± 2.84

## Data Availability

The data shown in this research are available on request from the corresponding author.
